# Atomic-Scale Characterization of 180° Conductive Domain Walls in PbZr_0.1_Ti_0.9_O_3_

**DOI:** 10.1021/acsami.4c11565

**Published:** 2024-11-19

**Authors:** Panagiotis Koutsogiannis, Felix Risch, José A. Pardo, Igor Stolichnov, César Magén

**Affiliations:** 1 Instituto de Nanociencia y Materiales de Aragón (INMA), CSIC-Universidad de Zaragoza, 50009 Zaragoza, Spain; 2 Departamento de Física de la Materia Condensada, Universidad de Zaragoza, 50018 Zaragoza, Spain; 3 Nanoelectronic Devices Laboratory (NanoLab), Ecole Polytechnique Fédérale de Lausanne (EPFL), 1015 Lausanne, Switzerland; 4 Departamento de Ciencia y Tecnología de Materiales y Fluidos, Universidad de Zaragoza, 50018 Zaragoza, Spain; 5 Laboratorio de Microscopías Avanzadas, Universidad de Zaragoza, Campus Río Ebro, 50018 Zaragoza, Spain

**Keywords:** conductive domain walls, ferroelectrics, Pb(Zr,Ti)O_3_, epitaxial films, scanning transmission electron microscopy, electron energy loss spectroscopy, nanoelectronics

## Abstract

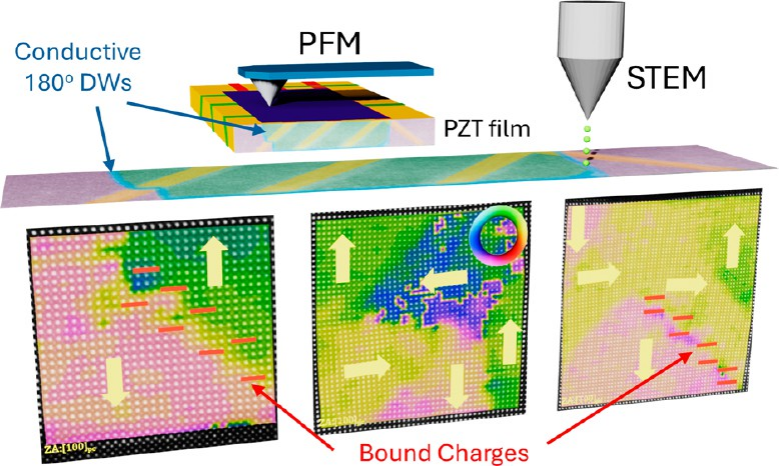

Conductive domain walls (DWs) in ferroic materials have emerged as promising candidates for applications in nanoelectronics due to their unique properties such as high conductivity and nonvolatility. In this study, we investigate the atomic structure and conductivity of nominally neutral 180° DWs artificially created in an epitaxial thin film of tetragonal PbZr_0.1_Ti_0.9_O_3_. Using piezoresponse force microscopy and scanning transmission electron microscopy, we elucidate the complex structure of these 180° DWs and their coupling with ferroelastic domains, revealing that they exhibit a complex structure due to the strain-mediated interplay with the ferroelastic domains. Our results demonstrate that the 180° DWs conductivity is associated with the emergence of polar discontinuities, including the formation of tail-to-tail charged segments, which has been further confirmed by electron energy loss spectroscopy. Additionally, we investigated the long-term performance of these domain boundaries, demonstrating their unique mobility and structural stability. Our findings provide insights into the atomic-scale mechanisms that turn nominally neutral DWs into highly conductive channels, paving the way for their use in advanced nanoelectronic devices.

## Introduction

Domain walls (DWs) in ferroic materials are being extensively studied aiming to understand the fundamental physics behind their unique properties, which are different from those of the domains themselves.^1−4^ Conductive DWs formed in insulating materials are a particularly exciting class of DWs for their unexpected behavior and potential for applications. Their ability to conduct electricity up to several orders of magnitude^5^ higher than the host material, makes them archetypal functional elements for future devices in the realm of nanoelectronics.^6^ Furthermore, their unique plasticity^7^ and nonvolatility^8^ are key ingredients for their application in memory storage and neural networks. In addition, DWs in ferroelectric materials can be tuned through the application of external electric fields modifying their shape, length, and chemistry and thus their resistance. Furthermore, in the case of ferroelectric thin films, strain,^9,10^ thickness,^11^ and different types of domains might alter the physics of the DWs.

Experimental demonstrations of nonvolatile conductive DWs in BiFeO_3_,^12^ PbZr_0.2_Ti_0.8_O_3_,^13^ PbTiO_3_,^14^ or LiNbO_3_^15^ have shown that perovskite ferroelectric crystals are ideal candidates for reconfigurable conductive networks. Electrical conduction in a wall between two ferroelectric domains requires the presence of bound charges at the boundary, often induced by an abrupt change of polarization, which is screened by mobile charges. Conductivity in charged DWs of different materials can have either an intrinsic or extrinsic origin. For instance, 90° DWs can bend and introduce polar discontinuities which lead to charged DWs with nonthermally activated intrinsic conductivity.^16^ Conductivity of 180° DWs,^13^ which ideally exhibit no bound charges, has been extensively studied. For instance, in Pb(Zr,Ti)O_3_ the presence of bound charges is predominantly influenced by extrinsic factors, such as the presence of oxygen vacancies and defects.^17^ DW tilting with respect to the polarization orientation,^18^ which can induce tail-to-tail (or head-to-head) polarization components, also induces a net bound charge at the boundary with a remarkable impact on their stability.^18,19^ On the other hand, in multiferroic BiFeO_3_ an increase in carrier density and a decrease of the band gap width within the DW have been reported.^4^ Consequently, in tetragonal Pb(Zr,Ti)O_3_ thin films, both extrinsic and intrinsic mechanisms might introduce new means for electrical conductivity. However, disentangling the individual impact of the different mechanisms contributing to conductivity of materials in sharp, atomic-thick boundaries such as DWs is a formidable task, which requires a detailed knowledge of the atomic-scale structure of conductive DWs.

Here we focus on the atomic structure characterization of artificially created nominally neutral 180° DWs in a tetragonal PbZr_0.1_Ti_0.9_O_3_ (PZT) thin film epitaxially grown on DyScO_3_, with a conducting SrRuO_3_ buffer layer. Using scanning transmission electron microscopy (STEM), we investigate the complex DW structures, with atomic resolution, to explain the origin of conductivity in the stable, nominally neutral DWs reported by Risch et al.^2^ In this work, the metallic-like nonthermally conduction of the 180° DWs suggests that an intrinsic mechanism should dominate. For this reason, special attention is paid in this study to the affinity between the artificially created 180° DWs and the ferroelastic DWs originally formed in the pristine regions of the thin film due to strain. Their coupling is found to be essential for their unique structure and how their conductivity and plasticity can be exploited for memristive applications. Additionally, electron energy loss spectroscopy (EELS) measurements show evidence of unprecedented changes of the local electronic structure of the artificial nominally-neutral 180° DWs, which can explain the high conductivity of the DWs.

## Methods

### Thin Film Growth

An epitaxial PZT film with tetragonal structure (bulk lattice parameters:^20^*a* = 3.904 Å, *c* = 4.135 Å) was grown on single-crystalline, (110)-oriented DyScO_3_ (DSO) substrate from CrysTec GmbH, with the pseudocubic (pc) lattice parameters of *a*_pc_ = 3.943 Å, *b*_pc_ = 3.945 Å being in-plane, and *c*_pc_ = 3.943 Å out-of-plane (the relationship between the orthorhombic (o) and pseudocubic axes of DSO is [110]_o_//[001]_pc_, [001]_o_//[010]_pc_, [110]_o_//[100]_pc_). A conducting SrRuO_3_ (SRO) buffer layer was used as the bottom electrode. The small lattice mismatch between the film and the substrate promotes the epitaxial growth of PZT with the *c* axis preferentially aligned parallel to the [001]_pc_ direction of the substrate. This also leads to the emergence of some *a* domains with their long axis aligned parallel to either the [100]_pc_ or the [010]_pc_ directions of the substrate. The *a* domains appear distributed in a nearly periodic manner separating the larger *c* domains, thus minimizing strain^21^ and annihilating depolarizing fields.^22^ The DSO substrate used here presents a precise miscut angle of 0.1°, oriented along the [110]_o_ crystallographic direction. The film was grown by pulsed laser deposition (PLD) using a KrF excimer laser with a fluence set at 1 J/cm^2^. The substrate temperature was 625 °C for SRO and 575 °C for PZT, and the oxygen pressure was 0.145 mbar for SRO and 0.25 mbar for PZT. The sample was cooled at a rate of 15 °C/min under an oxygen pressure of 1 mbar. The thicknesses of the SRO and PZT layers, as determined by STEM cross-sectional imaging, were found to be 22.5 and 66.9 nm, respectively. The thickness of SRO was optimized in order to guarantee high conductance for the electrical measurements and to avoid the presence of islands and valleys appearing at lower SRO thickness.^23^ The thickness of PZT was chosen in order to obtain an increased number of *a* domains compared to other thicknesses,^11^ which results in an enhanced conductivity at the 90° DWs due to a slight bending from their neutral orientation.^16^

### Ferroelectric Characterization and Poling

Piezoresponse force microscopy (PFM) was performed by using an Asylum Research Cypher AFM system (Oxford Instruments) equipped with an environmental scanner. For the PFM and poling experiments, conductive boron-doped diamond-coated tips (AD-40-AS) with a 40 N/m stiffness and tip radius of 10 ± 5 nm from ADAMA Innovation were used. The PFM images were collected with a built-in dual AC resonance tracking (DART) technique to enhance the PFM signal and allow for faster image acquisition.

Ferroelectric domain switching was carried out in stripe pattern areas of 15 × 10 μm^2^, in a succession of rectangular regions oriented along the ⟨100⟩_pc_ or the ⟨110⟩_pc_ directions of the substrate. A positive bias (+5 V) was applied at the SrRuO_3_ electrode above the coercive voltage of PZT, while the conductive probe was grounded at 0 V and scanned over the designated areas of the PZT surface. Some of the upward-polarized *c* regions were then partially switched downward, thus creating 180° DWs between the as-deposited and poled regions. Since the mobility of the DWs is uncertain during and after the subsequent lamella preparation, and the newly formed domains may shrink with time, different poling patterns were performed with a distance between the switched domains varying between 1 μm and 200 nm. The vertical response PFM images of the PZT surface and STEM analysis of the nominally 180° DW created in the latter case are shown in Figure S1.

### Electrode Patterning

Top Cr/Au (5/20 nm) 2 × 2 μm^2^ electrodes were deposited on the PZT surface by using electron-beam lithography (Raith EBPG5000) and metal evaporation (Alliance-Concept EVA 760) together with lift-off techniques. The electrodes served as topological patterns and resembled the corners of 15 × 7.5 μm^2^ squares inside which the PFM poling patterns were done. The patterns also helped identify the poled regions during TEM sample preparation using the dual-beam microscope, as ferroelectric switching could not be directly traced.

### Scanning Transmission Electron Microscopy

Atomic characterization of the PZT domains and DWs was performed using a Thermo Fisher Scientific Titan 60-300 transmission electron microscope (TEM), operated at 300 kV. The microscope is equipped with a high-brightness Schottky field emission gun (X-FEG) and a Wien-filter monochromator, a CETCOR corrector for the condenser system to provide sub-Å resolution in STEM mode, and a Gatan imaging filter (GIF) Tridiem 866 ERS for electron energy loss spectroscopy (EELS).

High-angle annular dark field (HAADF) and annular bright field (ABF) imaging in STEM mode were used to capture both the cationic and oxygen sublattices simultaneously. Low-angle annular dark field (LAADF) imaging was used to highlight diffraction contrast associated with the presence of DWs. The convergence semiangle was set at 24.8 mrad using a 70 μm aperture, while the acceptance semiangles were 47.9, 22.0, and 12.2 mrad for HAADF, LAADF, and ABF, respectively. The images were collected using moderate beam current between 5 and 20 pA. Dedicated scripts were employed to collect sets of 10 consecutive HAADF and ABF images. These images were acquired with a short dwell time (<1 μs/pixel), realigned with subpixel resolution, and averaged to correct for residual spatial drift and reduce electronic noise.

The atomic displacements of Zr/Ti and O columns along the in-plane (Δ*x*) and out-of-plane (Δ*z*) directions with respect to the substrate plane were calculated by measuring the shift from the centrosymmetric position defined by the A sites of the perovskite structure (Pb). The initial positions of the atomic columns were found using a blob detection algorithm tracking intensity maxima in the image and then refined by applying successive center-of-mass and 2D Gaussian fits using the Atomap python package.^24^ Finally, detailed polar displacement maps were constructed via the Temul package.^25^ In-plane and shear deformation maps were measured by applying geometric phase analysis (GPA) on HAADF-STEM images, to assess the local lattice deformation at the DWs.^26^

Electron energy loss spectroscopy in STEM was performed to assess the local changes in the electronic structure of the DWs. EELS spectra line scans were collected across the DWs with an acquisition time of 0.5 s per spectrum and a pixel size ranging from 0.2 to 0.4 nm. The electron beam was monochromated to obtain a zero-loss peak full width at half-maximum (fwhm) of 0.2 eV, and the GIF resolution was set at 0.05 eV. The convergence semiangle (α) was set at 17.7 mrad using a 50 μm aperture and the acceptance semiangle (β) was 27.2 mrad. The beam current varied between a few pA to hundreds of pA depending on the sensitivity of the sample to the electron beam.

Cross-sectional TEM lamellae were prepared in a Thermo Fisher Scientific Helios 650 dual-beam microscope. Different specimens were cut parallel to the [100]_pc_ and [110]_pc_ directions of the DSO substrate. The final thinning and polishing of the specimens were performed at low current (<7 pA) and voltage (5 kV) to minimize structural damage and DW motion due to heat dissipation and Ga^+^ bombardment.

## Results

**Figure 1:**
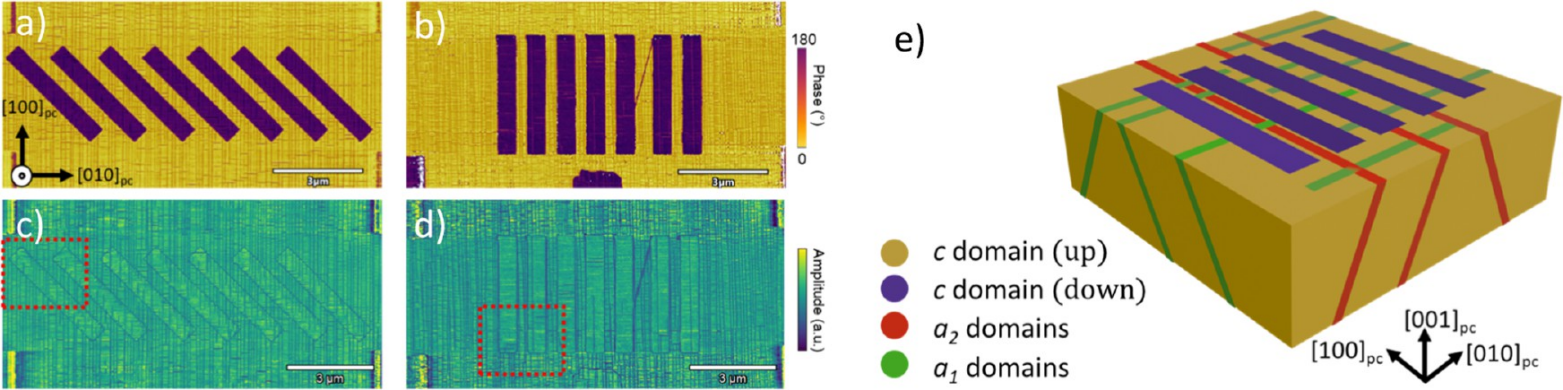
Vertical response PFM phase images of the PZT film after poling in rectangular regions oriented along the ⟨110⟩_pc_ (a) and ⟨100⟩_pc_ (b) directions of the substrate and their corresponding PFM amplitude images in (c) and (d). The stripe patterns, in dark purple in (a) and (c), indicate the poled regions which adopted the downward [001] polarization after poling, while the pristine film (in yellowish tones) was polarized upward [001]. The long bluish domains spanning along the [100] direction in the PFM amplitude images (c, d) highlight the existence of ferroelastic *a*_1_ domains along the [100] direction, while the short horizontal lines indicate the presence of ferroelastic *a*_2_ domains along the [010] direction. The dotted squares in (c) and (d) illustrate the regions where the closed-up images shown in Figure S3 were collected from. (e) 3D sketch of the PZT film with the poled stripe pattern along the ⟨100⟩_pc_ direction shown in (b) and (d). The pristine *c* domains are colored in yellow, the poled *c* domains in purple, and the two types of *a* domain (*a*_1_ and *a*_2_) are depicted in red and green, respectively.

Vertical response PFM images shown in Figure 1 illustrate the structure of ferroelectric domains at the surface of the PZT film after poling different regions of the film in rectangular patterns aligned parallel to the ⟨100⟩_pc_ and ⟨100⟩_pc_ directions of the substrate, as described in the Methods section. The distance between the poled domains is 350 nm. The pristine surface mostly consists of upward-polarized *c* domains, depicted with yellowish tones in the phase images of Figure 1a,b. The maroon rectangular regions correspond to the downward-poled *c*-domain areas. Thus, 180° DWs must form at the boundaries of the rectangular regions, as confirmed by the sharp dark blue contrast outlining the poled regions in the PFM amplitude images in Figure 1c,d. The PFM amplitude images also evidence a dense pattern of blue stripes parallel to [100]_pc_ and [010]_pc_, which are associated with the spontaneous formation of ferroelastic *a* domains in which the polarization of PZT lies approximately parallel to the substrate plane. These domains emerge as a consequence of the stress that develops between the film and the substrate due to their different in-plane lattice parameters, specifically because the pseudocubic lattice parameter of DSO is between the *a* and *c* lattice parameters of PZT. Their size, orientation, and abundance are determined by the complex interplay between the epitaxial strain and the depolarization field as a function of temperature and film thickness.^27^ Notably, the *a* domains are strongly suppressed in the poled regions, as shown in Figure 1d, which can be explained by the high electric fields developed during poling inducing drastic domain reconfigurations.^2^ Two different kinds of *a* domains are formed as a result of the slightly anisotropic epitaxial strain induced by the orthorhombic DSO substrate, the growth conditions,^11^ and the film thickness.^9^ First, plate-like regions (which we call *a*_1_ domains) reach the surface at long lines oriented along the ⟨100⟩_pc_ axis. Second, *a*_2_ domains produce shorter straight lines aligned parallel to the ⟨010⟩_pc_ directions.^28^ The whole scenario of the ferroelectric domains of the PZT film after poling is schematized in Figure 1e.

As explained before, nominally 180° DWs between *c* domains have no bound charges and are insulating. However, they have been shown to present nonthermally activated metallic-like conductance^2^ (see Figure S2, where the conduction around a two-square pattern is shown). To understand the domain structure induced by poling and investigate the origin of such DW conduction at the atomic scale, we first analyzed the domains themselves and, afterward, the shape of the DWs. Later we focused on atomic characterization and analysis of polarization at the DWs.

**Figure 2:**
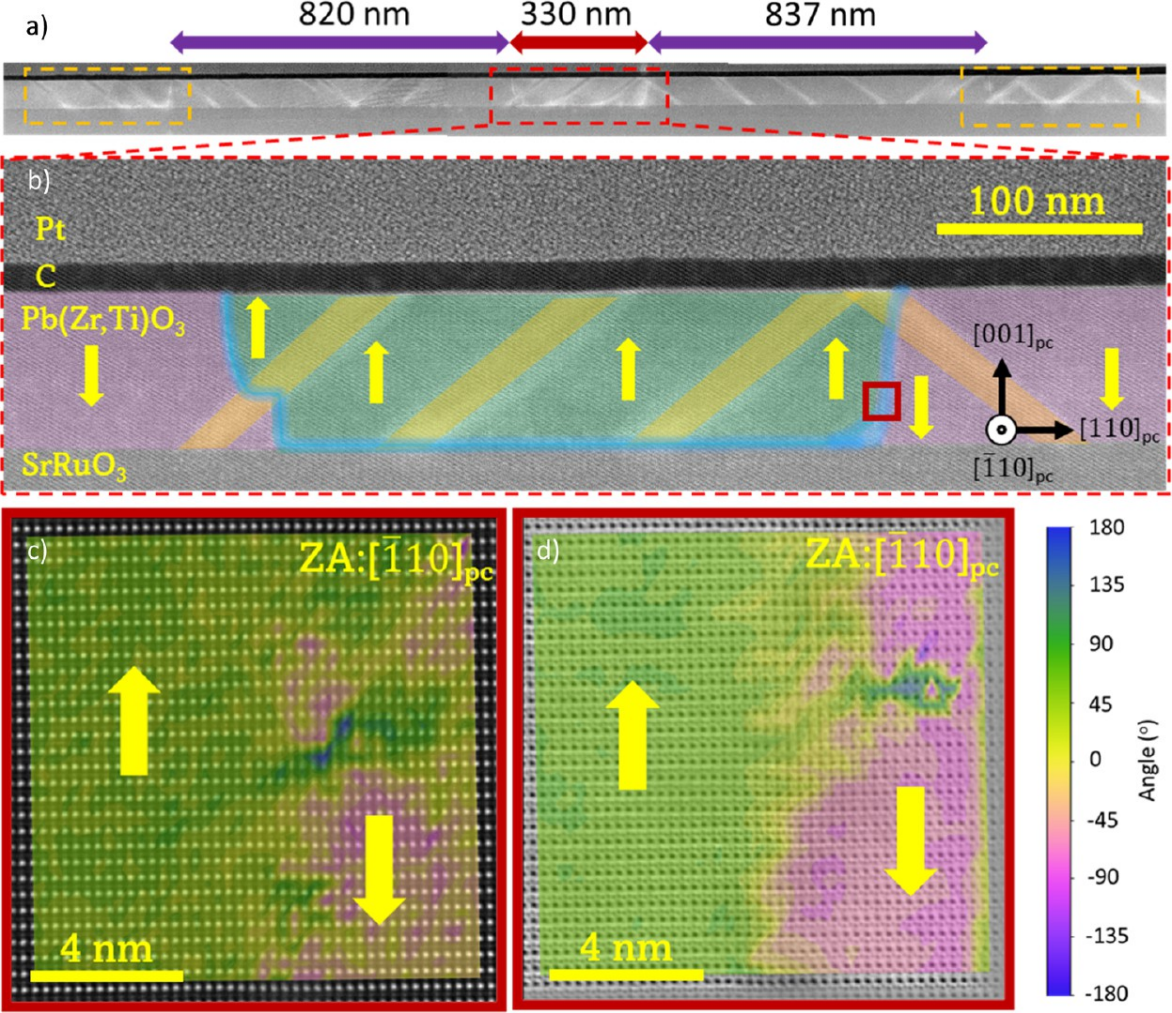
STEM images of the poled sample shown in Figure 1a,c. (a) Low magnification LAADF-STEM image of two consecutive poled regions, 820 and 837 nm wide, and a total of three pristine regions highlighted with dash lines. (b) A close-up image of the central region marks up the direction of polarization in the pristine (green), poled (pink), and *a* domains (yellow), as well as the nominally 180° DWs (blue). The arrows indicate the direction of polarization. (c) HAADF-STEM and (d) ABF-STEM images overlaid with a color map of the angle of polarization with respect to the horizontal substrate plane collected from the red square area indicated (b).

Figure 2a shows a LAADF image of the TEM specimen extracted from the sample shown in Figure 1a,c cut along the ⟨110⟩_pc_ direction, (with the zone axis parallel to the [110]_pc_ direction) and illustrates the distribution of *a* and *c* domains. Oblique *a*_1_ and *a*_2_ ferroelastic domains are apparent from the bright diffuse contrast associated with the local strain. A slightly brighter LAADF contrast also highlights the boundaries between the pristine regions and the poled areas. As can be seen in the close-up image of Figure 2b, the central pristine area (in green) is enclosed by two tracks of bright diffuse contrast (marked in blue) that run approximately vertically, corresponding to the expected 180° DWs, which are connected by an additional diffuse contrast line along the PZT-SRO interface. The fact that the poled area shows a diminished diffuse contrast at that interface is the indication of a noticeable local strain relaxation at the interface with the SRO electrode associated with the ferroelectric poling. The atomic-resolution HAADF and ABF images shown in Figure 2c,d were collected from the region highlighted with a red frame in Figure 2b. The HAADF image was used to analyze the displacement of the Ti/Zr columns, while the ABF image focused on the displacement of the O sublattice. This analysis visualizes the 180° DW between the *c* domains and facilitates the subsequent calculation of the polarization orientation, illustrated through the overlaid color maps.

On the contrary, a TEM specimen cut along the ⟨010⟩_pc_ direction shows only *a*_2_ domains that span along the [100]_pc_ direction of the substrate, perpendicular to *a*_1_ domains that span along the [010]_pc_ direction. The film is populated by two types of *a*_2_ domains, which we label *a*_2-I_ and *a*_2-II_ (hereinafter, *a*_I_ and *a*_II_), inclined along the (011)_pc_ and (011)_pc_ planes of tetragonal PZT, respectively.

To minimize the energy cost associated with bound charges at the DWs between the *c* and *a* domains in the case of an upward polarized *c* domain, polarization continuity is energetically favored. Consequently, the *a*_I_ and *a*_II_ domains polarization is expected to align along [010]_pc_ and [010]_pc_ directions, respectively.^29^

It has been reported that the *a*_I_ and *a*_II_ domains in PbTiO_3_ are coupled with *c* domains and create regions of superdomain structures, with a period of hundreds of nm.^30^ The close-up amplitude PFM image illustrated in Figure S3a shows the reorganization of *a*_1_ and *a*_2_ domains in the poled regions. More specifically, most of the *a*_1_ and *a*_2_ domains existing in the film are interrupted at the DWs of the newly formed switched regions, while only a few emerge. It is also evident that the reorganization of the *a*_1_ and *a*_2_ domains is highly dependent on the poling direction. Specifically when the poling patterns exhibit DWs parallel to the *a*_1_ and *a*_2_ domains, most of them disappear in the poled region, while a different pattern emerges when the DWs of the poled domains are oriented at 45° to the *a* domains. Remarkable changes of the *a* domain crystal orientation rather than a mere polarization switching have already been reported elsewhere.^2^ A two-step polarization switching leads to an intermediate state with *a*_1_ and *a*_2_ domains in the poled region, which vanishes with increasing bias.^28^

**Figure 3:**
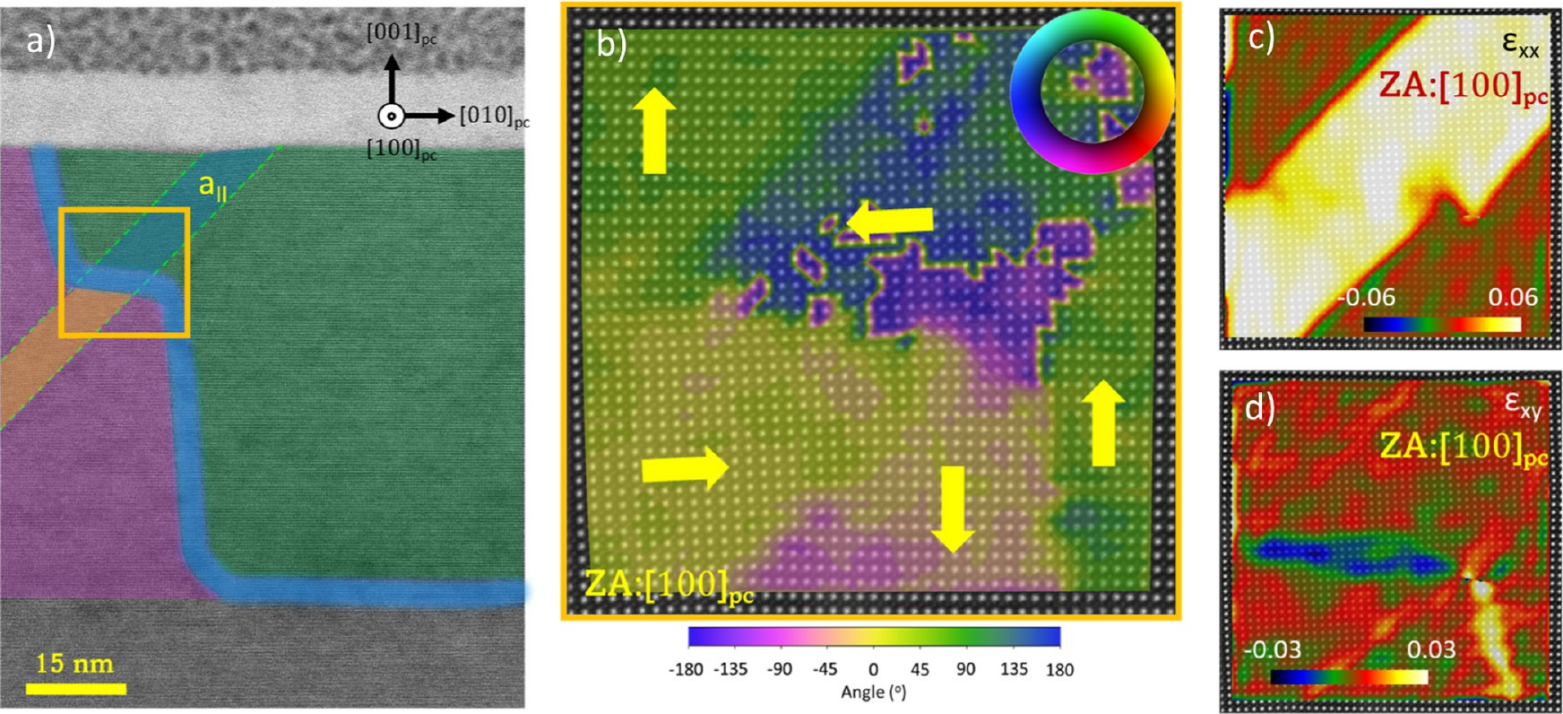
STEM images and GPA analysis of a nominally 180° DW crossing an *a*_II_ domain collected from the poled sample shown in Figure 1b,d. (a) Low-magnification ABF-STEM image. The 180° DW is marked in blue, the *a*_II_ domain is highlighted with dashed green lines, while the pristine region is colored in green and the area poled downward in pink. (b) Atomic-resolution HAADF-STEM image collected at the intersection of the 180° DW with the *a*_II_ domain, marked with a yellow square in (a), overlaid with a color map of the angle of polarization that features the polarization switching of the bottom *a*_II_ domain. (c) In-plane (ε_*xx*_) and (d) shear (ε_*xy*_) GPA deformation maps evidencing the lattice strain induced by the polarization switching of the ferroelastic *a*_II_ in the poled region with respect to the pristine area.

Unlike the emergence and disappearance of *a*_1_ and *a*_2_ domains with increasing bias, our TEM analysis shown in Figure 3 of the specimen extracted from the sample shown in Figure 1 (b,d), cut along the ⟨010⟩_pc_ direction, (with the zone axis parallel to the [100]_pc_ direction), confirms a different behavior. PFM poling induces a polarization switch from [001]_pc_ to [001]_pc_ in the *c* domains while also initiating polarization switching in the *a* domains. Between the poled and pristine regions of the *c* domains, an artificial 180° DW is created. However, the boundary between the poled and the pristine areas is often in close proximity to an *a* domain due to their abundance in the film, so when the new 180° DW intersects it, a new type of domain boundary is formed. In the case shown in Figure 3a, from the film surface to the substrate, such a 180° DW descends vertically along the *c* domain, rotates approximately 90° upon intersecting an *a*_II_ domain, and finally rotates back again to its original vertical descent to the buffer layer. The polarization analysis of the HAADF image in Figure 3b indicates that the ferroelastic *a*_II_ domain splits into two differently polarized regions, with polarizations along [010]_pc_ and [010]_pc_ at the top and bottom domains, respectively. Although the leftward polarization determined in the top *a*_II_ domain is inhomogeneous, a similar effect was reported by Gao et al.;^31^ it is evident that the polarization of the bottom *a*_II_ domain has necessarily switched rightward to preserve polar continuity at the DW.^32^ GPA traces the DW position due to the lattice distortion across the different boundaries upon poling. The variation of the different lattice parameters is highlighted by the in-plane *ε*_*xx*_ deformation map shown in Figure 3c by using the pristine *c* domain lattice as a reference. The modification of the lattice distortion at the DW is highlighted by the ε_*xy*_ shear component map shown in Figure 3d. The emergence of shear strain at the DW can be explained by the displacement of the Pb sublattice in the poled domain, which allows polarization switching to occur. The DW shown in Figure 3 shows no tail-to-tail or head-to-head DWs, as polarization switching inside the *a* domain aids to preserve polar continuity and minimize the DW energy.

**Figure 4:**
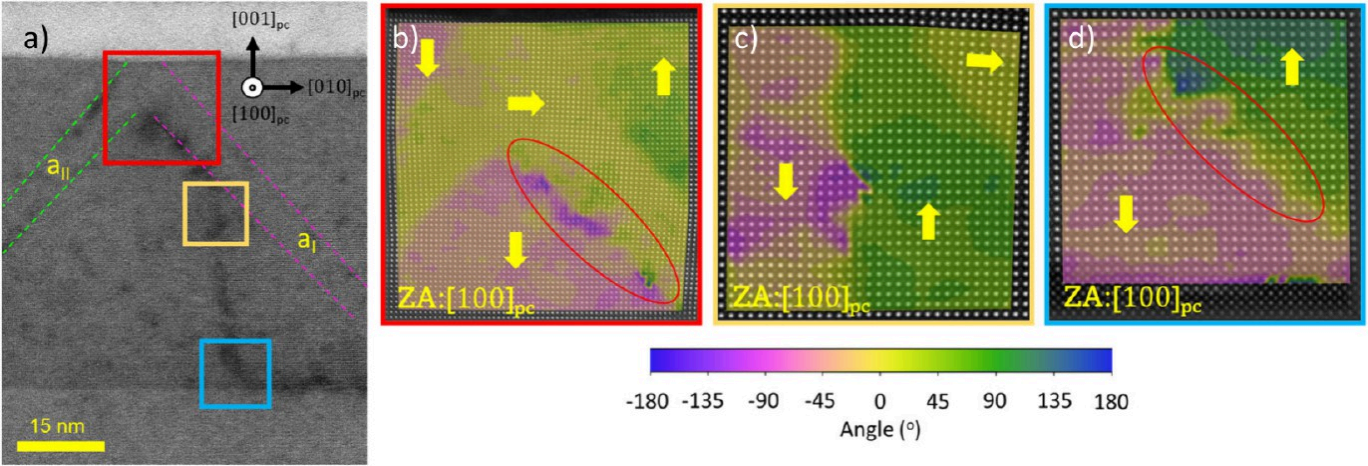
STEM images analysis of the nominally 180° DW crossing an *a*_I_ domain. (a) Low-magnification ABF-STEM image. The *a*_II_ domain and a neighboring *a*_I_ domain are highlighted with green and pink dashed lines, respectively. (b–d) HAADF-STEM images overlaid with a color plot of the polarization vector orientation in the vicinity of the nominally 180° DW, collected from the squared areas indicated in red (b), yellow (c), and blue (d). The polarization discontinuity at the DWs is highlighted with a red oval in (b) and (d).

On the contrary, when the artificial 180° DW encounters the bottom boundary between an *a* and *c* domain, it follows a different path compared to the case of the *a*_II_ domain shown in Figure 3. In this case, the *a* domain acts as a pinning region for the propagation of the 180° DW and hinders the switching of the subsequent *c* domain. This effect has been consistently observed across multiple regions and samples, as shown in Figure S4, confirming the robustness of the phenomenon. The out-of-plane electric field favors the polarization switching of the *a* domain from in-plane to out-of-plane, a process that requires the modification of the crystal lattice and is energetically unfavorable at low electric fields.^31^ STEM images, collected from the same sample shown in Figure 3 and presented in Figure 4a,b, show that the *c* domain above the *a*_II_ domain and the *c* domain beneath the top part of the *a*_I_ domain are fully switched. Focusing on the interconnection of the two *a*_I_ and *a*_II_ domains, we see that the top left and bottom *c* domains are polarized downward while the top right *c* domain is polarized upward. The analysis of the polar displacements provides evidence that both *a* domains stabilize with the rightward polarization orientation, giving rise to a polar discontinuity at the topmost boundary between the *a*_I_ domain and bottom *c* domain, enclosed in a red line in Figure 4b. This uncompensated bound charged segment with tail-to-tail configuration is energetically unfavorable, and the screening of bound charges could heavily contribute to the conduction of the artificial nominally 180° DWs.

Moving further downward, the boundary of the poled region emerges from the *a*_I_ domain to form a slightly tilted 180° DW as a consequence of inhomogeneous field in PZT and built in fields at the electrodes.^18^ The atomic shifts near this type of DW were analyzed in the HAADF (Ti and Zr) and ABF (O) images and are shown in Figure S5. As shown in Figure 4c, this 180° DW runs almost parallel to the (001) plane, separating two differently polarized *c* domains, and finally inclines along (001) only ∼7 nm above the interface with the SrRuO_3_ electrode. This inclination generates another tail-to-tail segment, shown in Figure 4d, which may attract screening charges and contribute to enhanced DW conductivity. A possible explanation for this unexpected inclination is that an *a*_I_ domain previously existed at this location where the 180° DW twist occurs, but it disappeared due to the strong electric fields applied during poling. The residual stress found at this position of the former *a*_I_ domain twists the DW parallel to the (110) plane. This explanation is supported by the phase-field simulations reported by Risch et al.,^2^ which show the twist of a 180° DW near the interface. In addition, more complex DWs are observed in the specimen shown in Figure 1c by imaging the oxygen lattice using ABF-STEM. The DW defined by the oxygen sublattice follows a slightly different trajectory from that of the Pb cations creating a complex DW with head-to-head and tail-to-tail regions. These complex DWs are shown in Figure S6 and further discussed in the Supporting Information.

**Figure 5:**
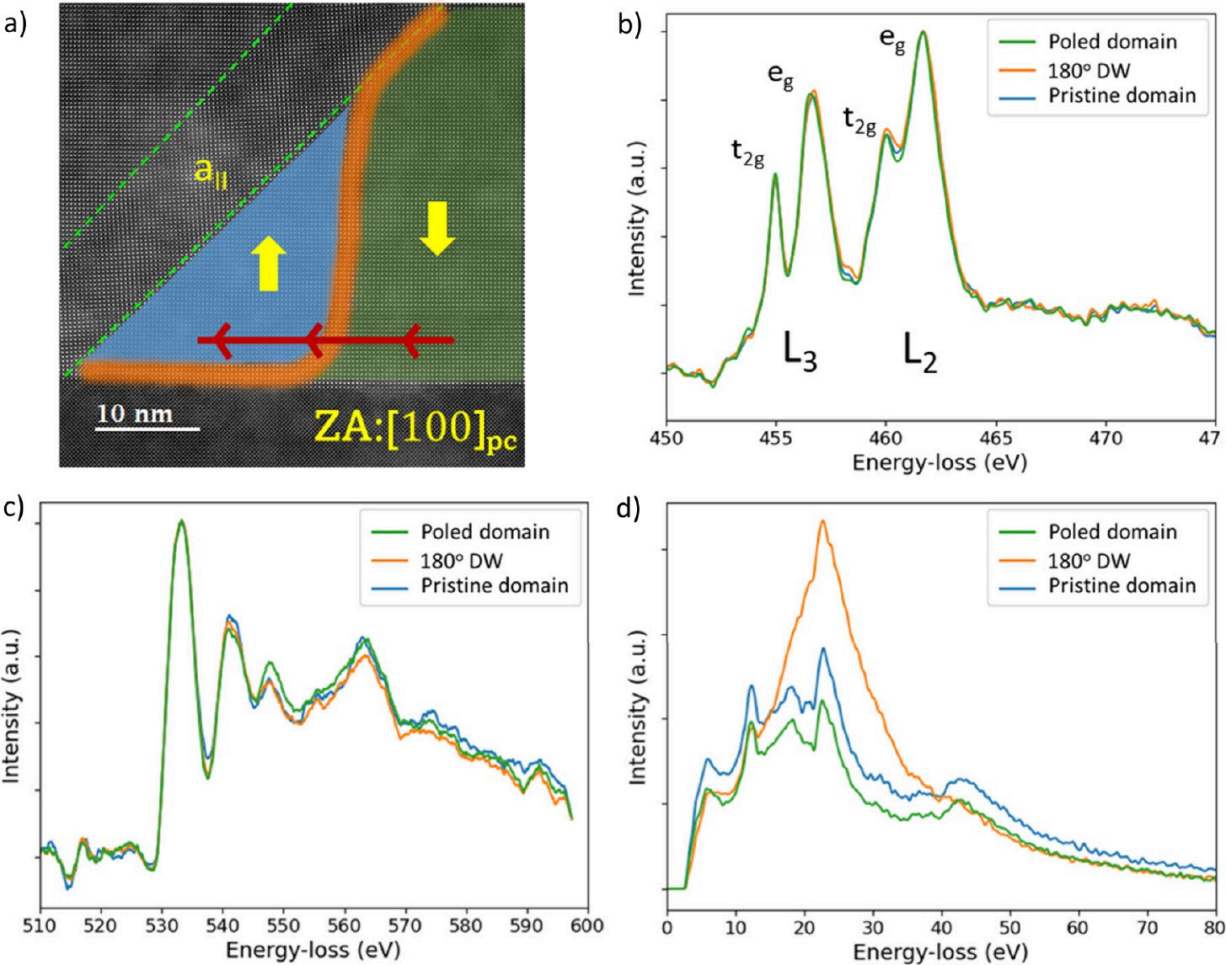
STEM-EELS analysis of the local electronic structure of the nominally 180° DW of the PZT thin film. (a) HAADF reference image, where red arrows indicate the area where the EELS line profiles were collected. The DW is marked in orange separating the pristine and poled domains highlighted in green and blue, respectively. (b) Averaged Ti L_2,3_ spectra, (c) averaged O K edge spectra, and (d) low-loss spectra collected across the DW.

It has been reported that DW conductivity is closely related to the formation of oxygen vacancies and the local reduction of Ti^4+^ in ferroelectric titanates.^16^ For that reason, the local electronic structure of the DWs presented in Figures 3 and 4 was examined using STEM-EELS line profiles. The electron beam was scanned along the [010] direction starting from the poled domain, crossing the 180° DW and ending in the pristine domain, as shown in Figure 5a. An EELS spectrum was collected every 0.125 nm. Given an approximate DW thickness of 2 unit cells, 8 spectra from each of the 3 different regions (DW, poled, and pristine domains) were summed and are illustrated in Figure 5b,c. Figure 5b illustrates the Ti L_2,3_ edge collected across the bottom side of the 180° DW shown, where bound charges are expected. The peaks of Ti L_2,3_ edge at the domain wall reveals no substantial energy loss differences (|Δ*Ε*(e_g,_t_2g_)| < 0.2 eV, arbitrarily positive or negative) or substantial intensity variations when compared to the pristine or poled domains. Furthermore, the O K edge fine structure shown in Figure 4c does not show any significant variation. This suggests that possible chemical variations at the domain walls are extremely subtle to quantify it, beyond the detection limits of this technique.

Remarkably, low-loss EELS spectra were collected in the same region and are depicted in Figure 5d. The pristine and poled PZT domain regions present numerous features between 5 and 22 eV, which are attributed to interband transitions from occupied valence bands and shallow core levels to the unoccupied conduction band.^33^ For instance, the peaks at 11 and 17 eV are attributed to the transitions from the O-2p to Ti-3d and Pb-6p levels, respectively. Interestingly, the peak at 22 eV, which has been assigned to the energy of the volume plasmon ℏω_p_,^34^ exhibits a notable increase of intensity at the DW with respect to either the pristine or the poled domains, which might be caused by the same local changes in the band structure due to the Ti reduction and the increased DW conduction.

**Figure 6:**
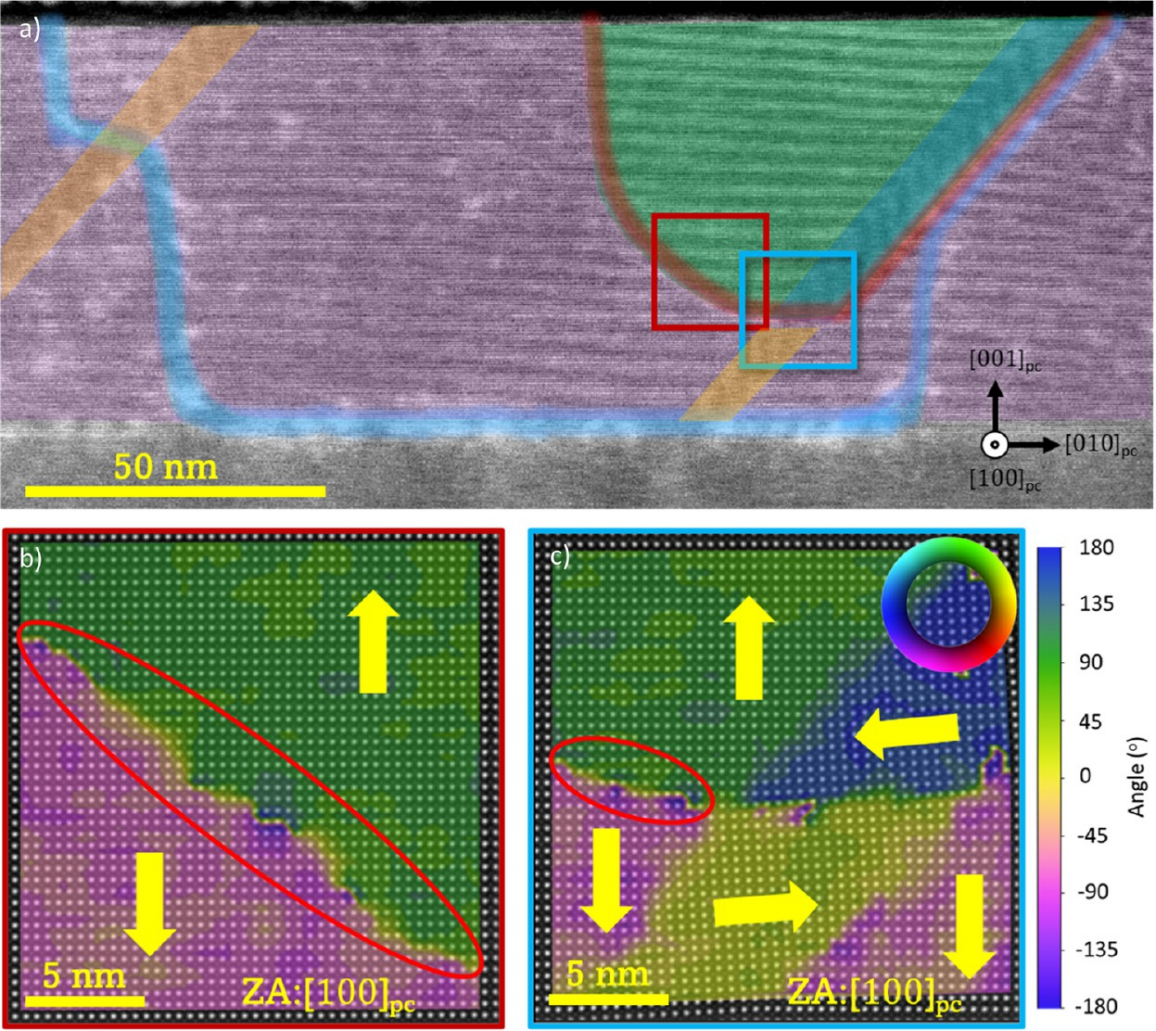
(a) LAADF-STEM image of the same region shown in Figure 3, after a 9 month storage. The initial position of the DW is observed and marked in blue, while the red line highlights the current position of the migrated DW. The green and pink areas indicate the upward and downward polarized domains, respectively. (b, c) HAADF-STEM images overlaid with a color plot of the polarization vector orientation in the vicinity of the nominally 180° DW, collected from the squared areas indicated in red (b) and blue (c).

Finally, we investigated the stability of the DWs after storing the lamellae in a desiccator for 9 months. The nominally 180° DW shown in Figure 3 attached to the *a*_II_ domain migrated toward the next *a*_II_ domain to the right, reducing the size of the pristine domain. As can be seen in Figure 6a, while the DW migrated to a new position (marked in red), the diffused contrast originated by strain remains at the initial position of the DW after poling, highlighted in blue. The new position of the migrated 180° DW exhibits no strain, but its curved geometry gives rise to new regions of polarization discontinuity. The top part of the migrated 180° DW shows a Néel-like rotation similar to that demonstrated by De Luca et al.^35^Figure 6b,c shows that the new DW is atomically flat and polarization discontinuity is constrained between two adjacent unit cells. Polarization continuity is restored when the nominally 180° DW impinges the *a*_II_ domain, and the bottom part of the *a*_II_ domain switches polarization as part of the process to minimize the DW energy, similar to the *a*_II_ domain shown in Figure 3.

## Conclusions

Our investigation provides a detailed examination of the atomic structure and electrical behavior of nominally neutral 180° DWs in a tetragonal PZT thin film. Utilizing atomic-resolution STEM, we conducted a thorough analysis of the DWs’ morphology and composition. Our findings highlight a complex interplay between the 180° DWs and ferroelastic domains, driven by strain effects and leading to the emergence of polar discontinuities and lattice distortions at the domain boundaries, which are crucial factors influencing their electrical conductivity. Moreover, EELS measurements provided valuable insights into the charge distribution within the DWs, confirming the presence of charges associated with high DW conductivity. This experimental evidence demonstrates that the nominally 180° DWs have a more complex inner structure with charged segments and supports the hypothesis that charged DWs serve as conductive pathways in PZT thin films. Furthermore, our long-term storage study demonstrated the slow mobility and resilience of DWs, suggesting their potential for practical applications in nanoelectronics. By elucidating the underlying mechanisms governing the electrical properties of DWs on the atomic scale, our research could contribute to the development of novel memristive devices and reconfigurable circuits. Overall, our study lays the foundation for harnessing the unique properties of DWs in PZT thin films for advanced electronic applications.

## Supporting Information Available

Additional experimental details on ferroelectric poling of the PZT film; electrical characterization of the nominally neutral 180° DWs by conductive AFM; PFM images of the reorganization of the *a* domains after poling; STEM images illustrating the interaction between 180° and 90° DWs; details of the determination of atomic displacements around the DWs; STEM analysis of a twisted DW (PDF)
