# Supplementary material for: Atomic-Scale Characterization of 180° Conductive Domain Walls in PbZr0.1Ti0.9O3
Source: ACS Appl Mater Interfaces. 2024 Nov 19;16(48):66341–9. doi: 10.1021/acsami.4c11565 (PMC11622216; doi:10.1021/acsami.4c11565)
Supplement: Supplementary file 1 — am4c11565_si_001.pdf [file am4c11565_si_001.pdf]

Supporting information

Atomic-scale characterization of 180 degrees conductive domain walls in $PbZr_{0.1}Ti_{0.9}O_3$

Panagiotis Koutsogiannis[1,2], *Felix Risch*[3], *José A. Pardo*[1,4,5], *Igor Stolichnov*[3] and *César Magén*[1,2*]

1. Instituto de Nanociencia y Materiales de Aragón (INMA), CSIC-Universidad de Zaragoza, 50009 Zaragoza, Spain

2. Departamento de Física de la Materia Condensada, Universidad de Zaragoza, 50018 Zaragoza, Spain

3. Nanoelectronic Devices Laboratory (NanoLab), Ecole Polytechnique Fédérale de Lausanne (EPFL), 1015 Lausanne, Switzerland

4. Departamento de Ciencia y Tecnología de Materiales y Fluidos, Universidad de Zaragoza, 50018 Zaragoza, Spain

5. Laboratorio de Microscopías Avanzadas, Universidad de Zaragoza, Campus Río Ebro, 50018 Zaragoza, Spain

*Corresponding author: cmagend@unizar.es

1. Ferroelectric poling

Ferroelectric domain switching in $Pb(Zr,Ti)O_3$ (PZT) was achieved via poling by applying 5V to the $SrRuO_3$. At the boundary between the poled and pristine ferroelectric *c* domains, conductive 180° domain walls (DWs) emerge. To study the crystal structure and chemistry of these DWs, a succession of stripe patterns was carried out in designated areas on the PZT film. The spacing between the stripe patterns was varied between 1 μm and 200 nm to achieve well-separated 180° DWs while also maintaining close proximity between them to facilitate as many as possible inside the TEM specimen. Since the mobility of the DWs is thermally activated[1], samples with 1 μm nominal spacing demonstrated a spacing of ~330 nm between the stripe patterns when examined with TEM. However, in the case of the stripe pattern shown in Fig. S1 with a nominal spacing of 200 nm, the final spacing between the poled domains was only a few nanometers and in certain cases the DWs disappeared as full switching was achieved. Fig. S1 (c) shows a U-shaped DW where partial switching was achieved beneath it, while the remaining pristine domain is engulfed within the DW shown in blue. The polarization analysis in the vicinity of the DW shown in Fig. 1 (d) demonstrates that the area outside the U-shaped DW has fully switched while the area inside remains unchanged.

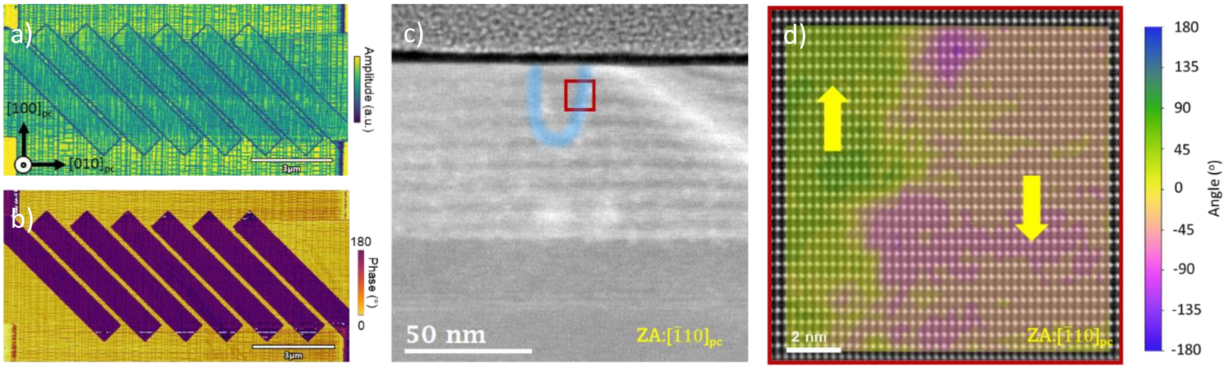

Figure S1: Analysis of the sample after poling with domains spaced nominally at 200 nm. PFM images show equally spaced poled domains and the formation of new *a* domains inside the poled region **(a,b)**. STEM-ADF image of the emerged DW, highlighted in blue, between the poled domains **(c)**. HAADF image overlayed with the polarization angle map collected from the region marked in (c), highlights the upward polarization of the pristine domain encapsulated between the U-shaped DW and the downward polarization surrounding the pristine domain **(d)**.

2. Electrical Characterization

Nominally 180º DWs emerge between ferroelectric *c* domains upon poling. These DWs have no bound charges and are insulating. However, they have been previously demonstrated to present non-thermally activated metallic-like conductance[2]. In this study, we aimed to analyze the nominally 180º DWs and understanding the origin of their high conductance. In this regard, ferroelectric poling was implemented on the PZT surface using 5 V at the $SrRuO_3$ electrode and scanning the AFM tip with 0 V bias, creating two square patterns shown in Fig. S2 (a). The emerged 180º DWs and 90º DWs at the boundary between *c* and *a* domains are highlighted in the amplitude PFM image in Fig. S2 (b). The conductivity of the DWs was unraveled using conductive-atomic force microscopy (cAFM) maps shown in Fig. S2 (c). The cAFM map acquired with 1.7 V tip bias features the high conductivity of the DWs that emerged upon ferroelectric poling. Last but not least, the surface of the PZT film examined by enhanced-resolution AFM (Fig. S2 (d)), shows no evidence of structural modification after ferroelectric poling.

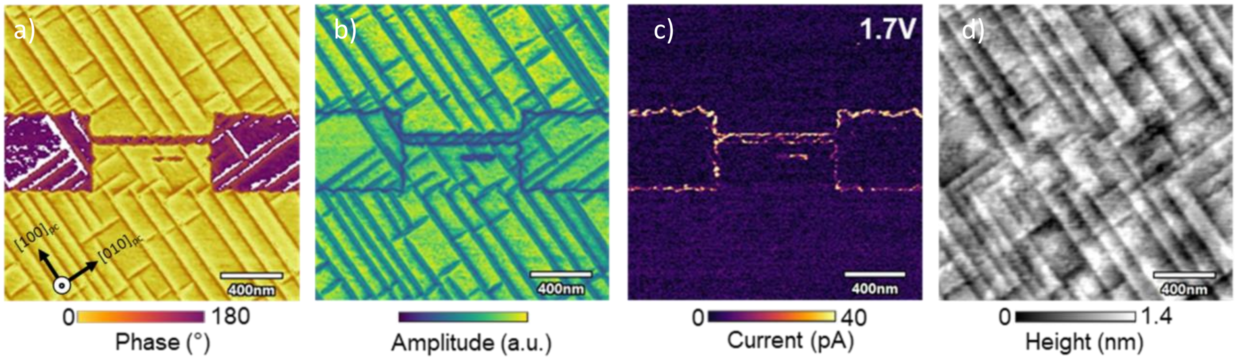

Figure S2: PFM- phase **(a)**, PFM- amplitude **(b)**, cAFM **(c)** and enhanced resolution topography **(d)** images of PZT surface with 180° DWs. PZT poling was implemented with the AFM-tip at 0 V bias and the $SrRuO_3$ electrode at 5 V. The two square patterns used for poling are visible in the phase image in (a). The topography image **(d)** shows minimal modification of the crystal inside the poled region. The amplitude PFM image (b) shows the emergence of a 180º DW at the boundary between the poled and pristine region. The conductivity of the formed DW is highlighted in the cAFM image (c).

3. *a* Domains Reorganization

Upon poling *a* domain reorganization occurs due to the emerging high electric fields. When poling stripe patterns toward the <100>$_{pc}$ direction, a significant reorganization of the a_1 and a_2 domains occurs. In the poled regions, many of the a_1 and a_2 domains that were present in the pristine film are interrupted at the newly formed DWs, while only a few *a* domains reemerge in different regions than before. This observation is well illustrated in Fig. S3 (a,b) The direction of the poling patterns strongly influences this behavior. Most of these domains disappear when the DWs in the poled regions are aligned parallel to the a_1 and a_2 domains. However, when poling stripe patterns towards the <110>$_{pc}$ direction, the DWs form at 45° to the *a* domains. This interaction differs, leading to distinct domain dynamics evidenced by the reemergence of a_1 and a_2 domains inside the poled pattern illustrated in Fig. S3 (d) in regions different than those demonstrated in Fig. S3 (c). The direct comparison between Fig. S3 (b) and Fig. S3 (d) shows that when poling extends towards the <100>$_{pc}$ direction a_1 and a_2 domains disappear and only a few reemerge in different regions inside the poled area. On the other hand, poling along <110>$_{pc}$ direction shows that a few of the a_1 and a_2 domains dominate while others disappear and reemerge and relocate in different regions inside the poled area.

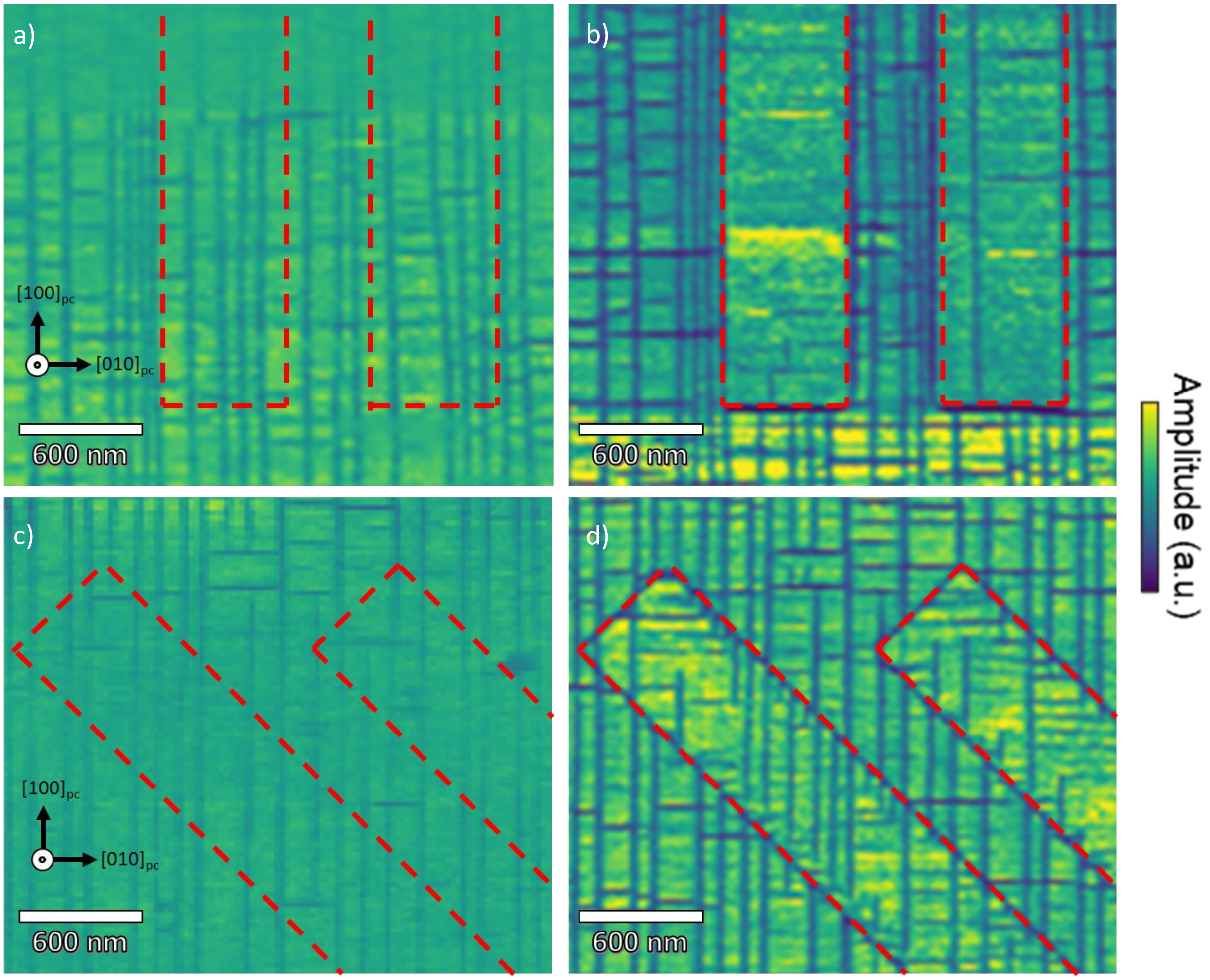

Figure S3: Close-up images of the amplitude PFM image that is shown in Fig. 1 (c,d), **(a,c)** before and after **(b,d)** poling. The red dush lines highlight the domain walls between the poled and pristine domain, marking the regions where the pristine *a* domains are interrupted and new *a* domains are formed.

4. Interaction between 180° and 90° Domain Walls

The interaction between 180° and 90° domain walls (DWs) has consistently demonstrated that when a 180° DW encounters the bottom boundary of an *a* domain, the latter acts as a pinning region, restricting the propagation of the 180° DW and preventing the switching of the subsequent *c* domain. Moreover, when a 180° DW encounters the top boundary of an *a* domain, it rotates approximately 90º crossing the *a* domain before it rotates back to its initial vertical position. These phenomena, demonstrated in Figs. 3 and 4, are not limited to a single instance, but they have been observed across multiple regions and different TEM samples, during our experiments. As evidence of the repeatability of these effects, additional examples of such 180°/90° DW interactions are provided in Fig. S4. These observations suggest that this interaction between 180° and 90° DWs is a robust phenomenon, at least in the studied material system.

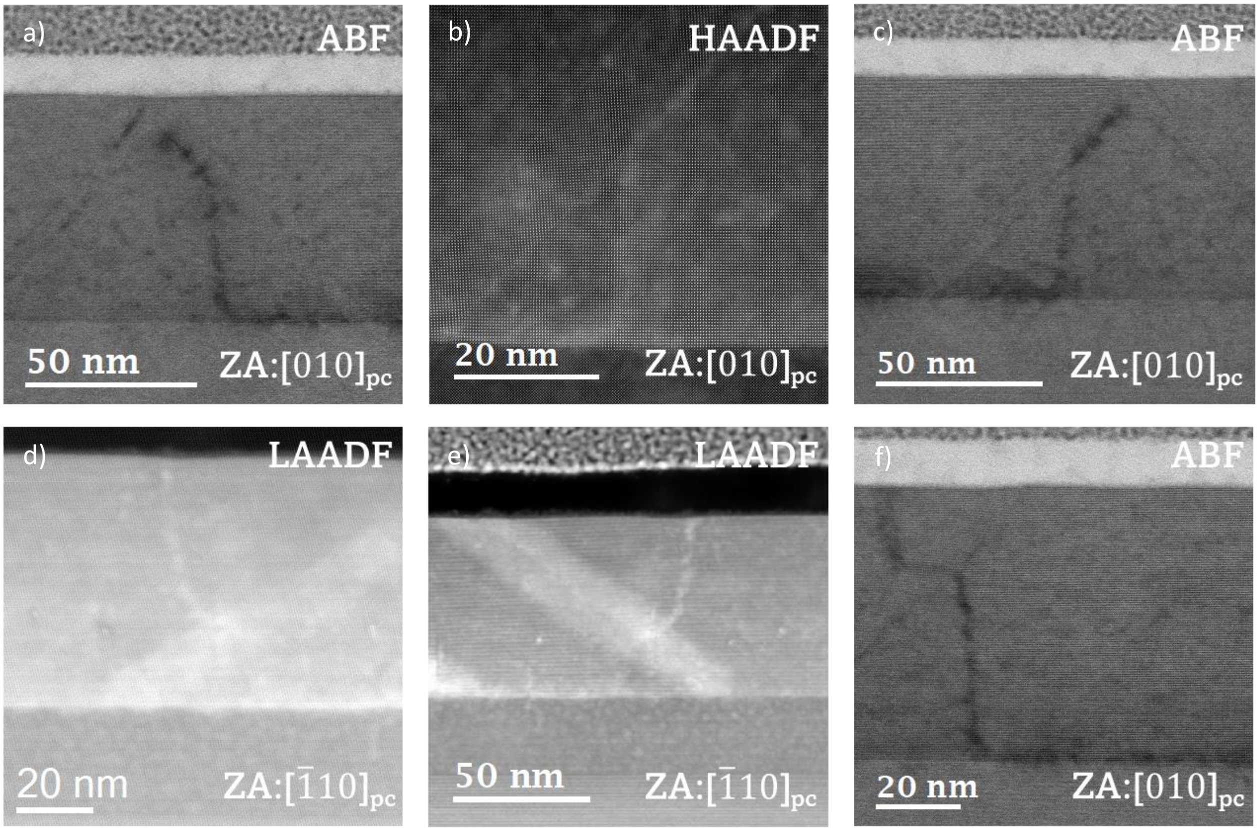

Figure S4: Various 180° and 90° DWs encounters observed during our experiments. The top panels show interaction between the DW and the bottom boundary of an *a* domain, where *a* domain acts as a pinning region, hindering the propagation of the 180° DW **(a-c)**. The bottom panels show interaction between the DW and the top boundary of an *a* domain, where the *a* domain is inoculated by the 180° allowing the switching of the subsequent *c* domain **(d-f)**.

5. Atomic displacements

The atomic shifts near the 180º DW were analyzed in the HAADF (Ti and Zr) and ABF (O) images and are shown in Fig. S5. The *c* domains of the pristine sample were polarized upward, while the poled *c* domains were polarized downward. The 180º DW close to the film's surface shows a slight outward bending of about 4º, towards the unpoled domain, indicating their weakly charged nature. The displacement of O anions from their centrosymmetric position is not equivalent in the two domains as shown in the ABF image in Fig S5 (b). The average O displacement in the poled and unpoled domain is -20 pm and 50 pm, respectively. The assessment of the atomic displacements of Ti suggests the vanishing of polarization within the 2 unit-cell-thick DW. The average Ti, Zr displacement along the [001] direction is 12.8 ± 2.1 pm in the poled and 9 ± 1.8 pm in the pristine domain. A detailed illustration of these displacements along the [001] direction can be seen in Fig. S5 (c). The difference in magnitude of the polar displacements between the two domains may be attributed to the imprint field which creates an asymmetry in the ferroelectric double well. Thus, the atomic displacement corresponding to the favored polarization state (downward) is stronger. The impact of the imprint field in the ferroelectric hysteresis loop (phase) and the amplitude of the cantilever are shown in Fig. S5 (d). In addition, minor crystal mistilt between the two regions might enhance the projected displacement in the two domains; ABF is particularly prone to inaccuracies in atomic displacement determination due to slight misalignments[3].

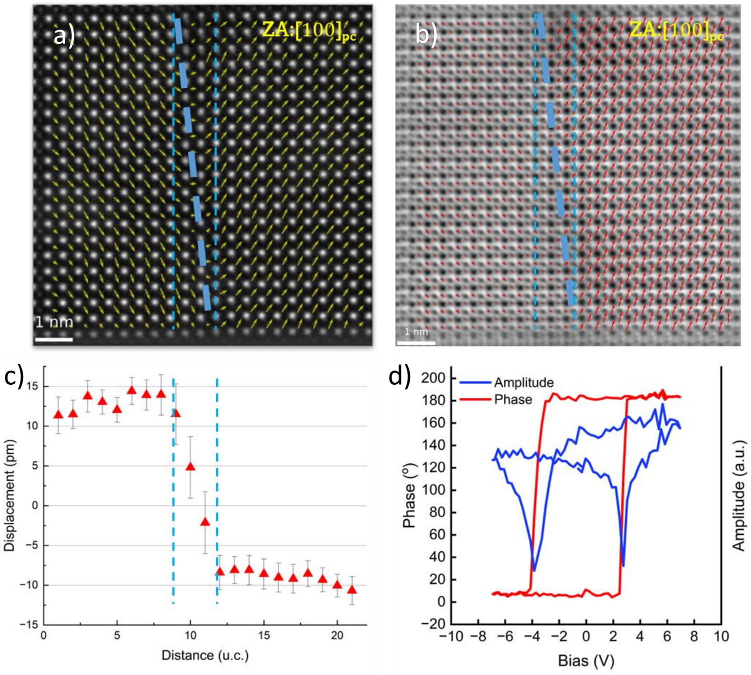

Figure S5: HAADF-STEM **(a)** and ABF- STEM **(b)** with their corresponding atomic displacement vectors overlaid. The direction of the domain wall in the two images is highlighted with a thick blue dashed line. The Ti, Zr displacement plot shows atomic shifts of about 12.8 pm and 9 pm in the poled and pristine regions, respectively **(c)**. Hysteresis loops of the phase (red) and amplitude (blue) signals as a function of bias voltage, measured via switching spectroscopy-PFM (ss-PFM). The butterfly-shaped curve of amplitude peaks at the coercive fields of -3.9V and 2.4V **(d)**.

6. Twisted domain wall

While 180º DWs appear straight or slightly bent along most of the *c* domain region of the films, interestingly the Ti and oxygen lattice are distorted twisting the DW repetitively making it look thicker and eventually broadening at the interface with the conducting SRO electrode as shown in Fig. S6 (a). The sudden rotation of the DW as revealed in the ABF image through O and Ti displacement analysis (Fig. S6, b and c), causes the local misalignment of the electric dipoles with respect to the domain boundary that is highlighted via the GPA ε_{xy} deformation map shown in Fig. S6 (d). In addition, the analysis of the Ti displacement in the HAADF image shown in Fig. S6 (e), shows a very similar behavior. The slight differences may be associated with the fact that the image is the projection of the twisted DW along the electron path, which may give rise to uncertainties in the determination of the atomic position similarly to the effect of mistilt or residual aberrations. This effect is usually more pronounced in phase-contrast ABF images than in HAADF[4]. The DW presents local head-to-head and tail-to-tail characters, thus becoming nominally charged, as reported elsewhere[5]. This type of DWs is usually screened by mobile charges, such as oxygen vacancies and electrons, that compensate for the polarization charges and create conductive channels inside the DWs themselves. Interestingly, these deformations of the 180º DW might be key to explaining the enhanced conducting properties of the DWs shown in Fig. S2.

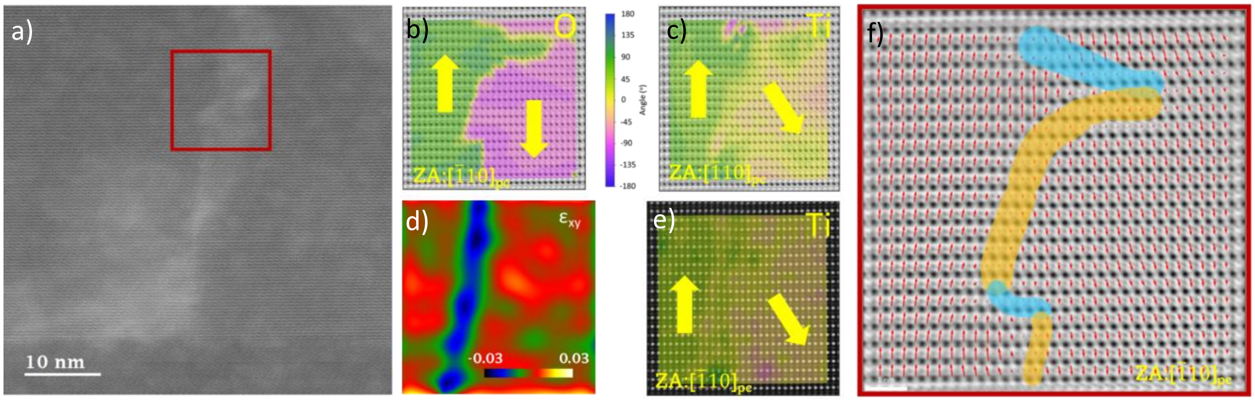

Figure S6: STEM images and GPA analysis of a twisted DW. STEM-ADF image of a nominally 180º DW **(a)**. STEM-ABF images of the DW collected from the region marked in (a), with the oxygen **(b)** and Zr, Ti **(c)** atomic displacement plot overlaid. ε_{xy} GPA deformation map highlights the shear strain of the Pb lattice which follows a different trajectory than that of the oxygen atomic displacements **(d)**. STEM-HAADF image of the DW collected from the region marked in (a) with the Ti atomic displacement plot overlaid **(e)**. The highlighted yellow and blue regions at the DW indicate areas with tail-to-tail and head-to-head DWs, respectively. The angle of the polarization vector indicates the disturbance and non-uniformity in the vicinity of the DW **(f)**.

References:

[1] Xu, F.; Trolier-McKinstry, S.; Ren, W.; Xu, B.; Xie, Z. L.; Hemker, K. J. Domain Wall Motion and Its Contribution to the Dielectric and Piezoelectric Properties of Lead Zirconate Titanate Films. *J. Appl. Phys.* **2001**, *89* (2), 1336–1348.

[2] Risch, F.; Tikhonov, Y.; Lukyanchuk, I.; Ionescu, A. M.; Stolichnov, I. Giant Switchable Non Thermally-Activated Conduction in 180° Domain Walls in Tetragonal $Pb(Zr,Ti)O_3$. *Nat. Commun.* **2022**, *13*, 7239.

[3] Zhou, D.; Müller-Caspary, K.; Sigle, W.; Krause, F. F.; Rosenauer, A.; van Aken, P. A. Sample Tilt Effects on Atom Column Position Determination in ABF-STEM Imaging. *Ultramicroscopy* **2016**, *160*, 110–117.

[4] Gao, P.; Kumamoto, A.; Ishikawa, R.; Lugg, N.; Shibata, N.; Ikuhara, Y. Picometer-Scale Atom Position Analysis in Annular Bright-Field STEM Imaging. *Ultramicroscopy* **2018**, *184*, 177–187.

[5] Jia, C. L.; Mi, S. B.; Urban, K.; Vrejoiu, I.; Alexe, M.; Hesse, D. Atomic-Scale Study of Electric Dipoles near Charged and Uncharged Domain Walls in Ferroelectric Films. *Nat. Mater.* **2008**, *7* (1), 57–61.
